# The increasing chronicity of HIV in sub-Saharan Africa: Re-thinking "HIV as a long-wave event" in the era of widespread access to ART

**DOI:** 10.1186/1744-8603-7-41

**Published:** 2011-10-20

**Authors:** Stephanie A Nixon, Jill Hanass-Hancock, Alan Whiteside, Tony Barnett

**Affiliations:** 1 Department of Physical Therapy, University of Toronto, 160-500 University Avenue, Toronto, ON, M5G 1V7, Canada; 2 Health Economics and HIV and AIDS Research Division (HEARD), University of KwaZulu-Natal, South Africa; 3 LSE Health, Department of Social Policy, London School of Economics, Houghton St, London WC2A 2AE, UK; 4 Department of Global Health and Development, London School of Hygiene and Tropical Medicine, 15-17 Tavistock Place, London WC1H 9SH, UK

## Abstract

HIV was first described as a "long-wave event" in 1990, well before the advent of antiretroviral therapy (ART). The pandemic was then seen as involving three curves: an HIV curve, an AIDS curve and a curve representing societal impact. Since the mid-2000's, free public delivery of life-saving ART has begun shifting HIV from a terminal disease to a chronic illness for those who can access and tolerate the medications. This increasing chronicity prompts revisiting HIV as a long-wave event. First, with widespread availability of ART, the HIV curve will be higher and last longer. Moreover, if patterns in sub-Saharan Africa mirror experiences in the North, people on ART will live far longer lives but with new experiences of disability. Disability, broadly defined, can result from HIV, its related conditions, and from side effects of medications. Individual experiences of disability will vary. At a population level, however, we anticipate that experiences of disability will become a common part of living with HIV and, furthermore, may be understood as a variation of the second curve. In the original conceptualization, the second curve represented the transition to AIDS; in the era of treatment, we can expect a transition from HIV infection to HIV-related disability for people on ART. Many such individuals may eventually develop AIDS as well, but after a potentially long life that includes fluctuating episodes of illness, wellness and disability. This shift toward chronicity has implications for health and social service delivery, and requires a parallel shift in thinking regarding HIV-related disability. A model providing guidance on such a broader understanding of disability is the World Health Organization's International Classification of Functioning, Disability and Health (ICF). In contrast to a biomedical approach concerned primarily with diagnoses, the ICF includes attention to the impact of these diagnoses on people's lives and livelihoods. The ICF also focuses on personal and environmental contextual factors. Locating disability as a new form of the second curve in the long-wave event calls attention to the new spectrum of needs that will face many people living with HIV in the years and decades ahead.

## HIV as a long-wave event

**Figure 1:**
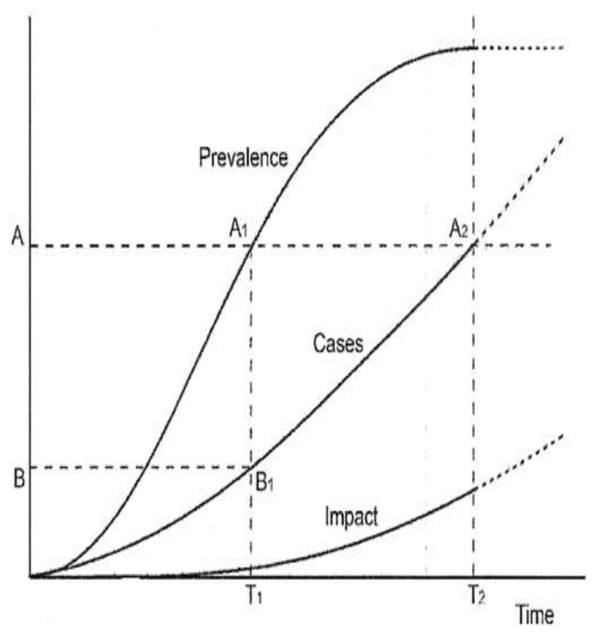
**The three HIV epidemic curves**.

"HIV as a long-wave event" was first described by Barnett and Blaikie in 1990 [1]. This idea was developed further by Barnett and Whiteside who conceptualized the long-wave nature of HIV in the three curves depicted in Figure 1[2]. In general, epidemics follow an S-curve (see the S-curve on the left in Figure 1). They start slowly and gradually. When a critical mass of infected people is reached, the growth of new infections accelerates (see the steep climb in the middle of the S-curve). The epidemic spreads through the population until all people who are susceptible have become infected. In the final stage of the epidemic (where the S-curve flattens at the top), people are either getting better or the number of deaths exceeds the number of new cases such that the total number of people living with the infection passes its peak and begins to decline. This decline typically occurs rapidly.

What sets HIV and AIDS apart from other epidemics is that there are additional curves to consider. The three curves in Figure 1 were conceived before the widespread availability of antiretroviral therapy (ART) [1,2]. First is the *HIV curve*, which was envisaged to precede a second curve, the *AIDS curve*, by 8 to 12 years. For the HIV curve, in the absence of a cure the only way to leave the pool of people with infections is by dying. The second curve, AIDS, reflected people who were becoming ill and, often, dying. The third curve represented *impacts*, which included orphaning, food insecurity and other societal concerns.

The innovation in this multi-curve approach was disaggregating the idea of a long-wave event into some of its constituent processes. This orientation drew attention to the need for long-term engagement in responding to the HIV epidemic. It also indicated an intergenerational problem because: (a) one outcome of the disease was increased orphaning as parents had children and then died prematurely, leaving those children to possible insufficient socialization, thus breaking the bond between generations; (b) inadequately socialized children were more likely to adopt risky sexual behaviours, thus replenishing the disease susceptible population age cohort [3]. Finally, this conceptualization of HIV as a long-wave event cautioned that most standard public health interventions for communicable diseases would be problematic given the ill-fit with funding streams and sheer magnitude of the problem.

## HIV in the era of expanded treatment access

Free public access to life-saving ART became available in parts of Africa in the mid-2000's, in contrast to many resource-rich countries where ART had been available from 1996. Despite this delayed start, by the end of 2009, 37% of people eligible for ART in sub-Saharan Africa were receiving treatment, compared with only 2% in 2002. As a result, AIDS-related deaths in Southern Africa dropped by almost one-fifth between 2004 and 2009 [4]. The advent of widespread access to ART in Southern Africa marks the dawning of a new era in the history of HIV as vast numbers of people living with HIV may expect to live far longer [5]. Indeed, the clinical, immunologic and virologic effects of ART for people living with HIV in resource-poor countries are well-documented [6-8]. Most people who can access and adhere to treatment can expect improvements in CD4 count and viral load, fewer opportunistic infections and overall reductions in HIV-related morbidity and mortality. HIV in high-prevalence, resource-poor countries is on the path toward becoming *a chronic illness *[9,10].

This increasing chronicity prompts revisiting HIV as a long-wave event. First, the advent of widespread ART means that the HIV curve will be higher and will last longer since people continue to become HIV-infected but are also living longer on treatment [11]. As a result, progression to AIDS on an individual level is far less predictable, although estimates can be made of treatment failure at a population level. The advent of better drugs at lower prices, especially second-line regimens, could further change the shape of the curve.

If patterns in sub-Saharan Africa mirror experiences in high-income countries, people on ART will live far longer lives but with new experiences of *disability *[12-18]. Disability, broadly defined, can result from HIV, its related conditions, and from side effects of the medications [19]. This shifting experience has stimulated innovative responses from rehabilitation, health and social sectors in many resource-rich countries [20-25]. However, it is likely that HIV-related disabilities in resource-poor settings will be more acutely disabling given the limited availability of rehabilitation, chronic health care services and social support grants.

Individual experiences of disability will vary greatly. At a population level, we anticipate that disability will become a common part of living with HIV, and may now be understood as a new version of the second curve. Whereas the second curve in the original conceptualization represented the transition to AIDS, in the era of treatment we can expect a transition from HIV infection to HIV-related disability for people who can access and tolerate ART. Many of these individuals may eventually transition to AIDS as well, but after a potentially long life that includes fluctuating experiences of illness, wellness and disability over time. This shift occurs in a milieu where increased resources are unlikely to be available in significantly greater quantities than they are now to support these elevated demands in terms of health infrastructure, rehabilitation and disability services, palliative care provision and/or medications to mitigate the effects of chronically disabling conditions.

## HIV-related disability

**Figure 2:**
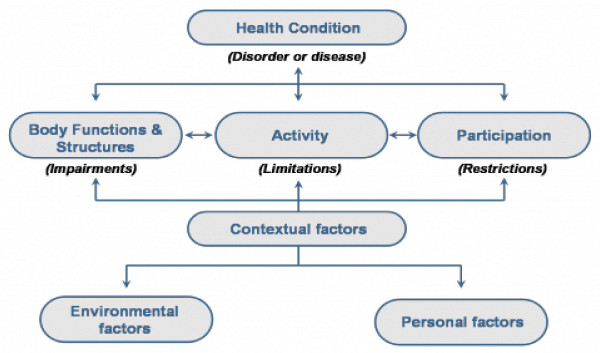
**The World Health Organization's International Classification of Functioning, Disability and Health (ICF)**.

ART has the potential to change HIV from a terminal disease to a chronic, albeit very serious, illness. This shift toward chronicity has significant implications for health and health care, and requires a parallel shift in thinking. How might we understand and anticipate the second wave of HIV-related disability? The word disability frequently invokes static and narrowly-conceived stereotypes. A broader understanding of disability as far-reaching and dynamic reflects a more realistic and constructive scenario. A widely-accepted model that provides guidance on such a broader understanding of disability is the World Health Organization's *International Classification of Functioning, Disability and Health (ICF*) (see Figure 2) [26,27]. The ICF describes diverse dimensions of human functioning affected by a health condition, including both biomedical and social concerns. In contrast to a biomedical approach that centres on diagnoses and symptoms, the ICF also focuses on the impact of these diagnoses at three levels: *body structure and function *(whereby *impairments *are challenges at the level of the body part or structure), *activity *(whereby *activity limitations *are challenges at the level of the whole person) and *participation *(whereby *participation restrictions *are challenges faced by the person in her environment or society). Challenges at all of these levels may be conceptualized as forms of disability. The ICF also understands personal and environmental contextual factors as shaping experiences at these three levels.

The ICF has been widely used in both resource-rich and resource-constrained settings for considering the disability dimensions of many health conditions [28,29]. Applied to HIV, impairments, activity limitations and participation restrictions can result from a diverse range of HIV-associated conditions affecting all body systems, including neurological and neurocognitive conditions resulting in brain or spinal cord problems, cardiovascular system changes resulting in strokes or heart attacks, musculoskeletal problems related to osteoarthritis and accelerated osteoporosis, and problems with vision or hearing. The strength of the ICF is its concern not only with these diagnoses, but with how these conditions affect people's lives and livelihoods. Disability resulting from HIV-related mental health conditions and neurocognitive changes [30] is also becoming better understood among people living with HIV in Africa, especially as it is pertains to elevated rates of depression [31]. Other mental health conditions with higher prevalence among people living with HIV in some African settings include bipolar disorder, schizophrenia, anxiety, post-traumatic stress disorder and sleep disorders [32]. Considerable disability can also result from the side effects of ART such as peripheral neuropathies linked to some medications, which can create pain and altered sensation in people's legs (impairment), potentially limiting their mobility (activity limitation), thus compromising their engagement in work or managing a household (participation restriction). Furthermore, the concept of "environmental contextual factors" within the ICF offers a link to the social, political and economic forces that may shape an individuals' experience of HIV-related disability, such as the profoundly important role of stigmatizing attitudes in creating and/or exacerbating disability.

The ICF has been used to conceptualize HIV in countries like Canada since the advent of ART in the late 1990's [23,33,34]; however, engagement with this framework for HIV in resource-poor settings has only recently begun. In 2009, Myezwa and colleagues used the ICF as the basis for a cross-sectional study that demonstrated a high level of disablement among 80 HIV-positive hospital inpatients [35] and among 45 HIV-positive outpatients in South Africa [36]. Even more recently, Myezwa *et al*. compared data from four cross-sectional studies (3 in South Africa, 1 in Brazil) that had applied the ICF as a classification instrument to people living with various stages of HIV and unequal access to ART [37]. Issues across all groups included weight maintenance and problems with sleep (50%, 92/185), energy and drive (45%, 83/185), and emotional functions (49%, 90/185). People on ART identified body image as a major problem. Other groups reported pain as a problem, and those with limited access to treatment also reported mobility problems. Cardiopulmonary functions were affected in all groups. Gaidhane *et al*. used the ICF to examine self-care among 194 people living with HIV in a tertiary care hospital in rural India, finding that over 65% of participants experiences one or more impairments [38]. This early evidence points to the spectrum of disability that we locate as the new second curve in the long-wave event of HIV.

## Looking ahead

The advent of ART in resource-poor settings has marked a dramatic shift in the epidemic giving rise to the potential onset of vastly elevated levels of HIV-related disability. Indeed, clinicians working in HIV may be familiar with patients whose clinical markers (e.g., CD4 count and viral load) indicate that they are doing well, yet they are struggling to manage. The reverse can also be true. This disconnect points to the importance of considering not only biomedical concerns (e.g., diagnoses, clinical markers, symptoms, drugs) but also the life-related impacts of HIV and its related conditions, which we term HIV-related disability. This shift also occurred in resource-rich countries in the 1990's upon the advent of treatment in those settings. However, the experience in Africa will be distinct in at least two important ways. First, the scope of the problem in terms of both absolute numbers and prevalence in many African countries dwarfs the experiences of many resource-rich countries. Second, the service delivery models for addressing disability-related concerns are stretched, fragile or non-existent in many African settings.

This analysis is reminiscent of the policy challenges flagged by Barnett and Blaikie in 1990 regarding the magnitude of the problem and the insufficient preparedness of the health system for responding to impending needs. Locating disability as a new form of the second curve in the long-wave event illuminates this concern today and in the future. It opens up new thinking about longer-term responses to these challenges in the era of ART. For example, reconceptualizing HIV using a disability lens highlights the need for engagement and education of many social sectors, some of whom may not as yet be engaged in the HIV response. This will include people involved in rehabilitation and/or disability efforts at the community, clinical practice, and policy levels [39]. The recently released World Report on Disability advocates for the adoption of the ICF as a universal framework for disability data collection across health conditions, offering a useful starting point as the HIV field shifts to consider HIV-related disability [27]. In terms of health systems, attending to the increasingly chronic nature of HIV offers links to existing efforts to recognize and address the increasing burden of chronic diseases [40]. Finally, national strategic plans to address HIV also need to take into account HIV-related disability and the diverse policy and programme responses required to address these coming changes in the experience of the disease [41]. Disability will affect many people living with HIV in the years and decades ahead, and concomitant responses from health, social and other sectors will be central to promoting health, quality of life and productivity.

## Competing interests

The authors declare that they have no competing interests.

## Authors' contributions

SN helped conceive the analysis, partially wrote the first draft and wrote the final draft.

JHH helped conceive the analysis, and wrote portions of the first and final drafts.

AW helped conceive the analysis, contributed to writing the first draft and critically reviewed the final draft. TB contributed to the analysis, and critically reviewed the first and final drafts. All authors read and approved the final manuscript.
